# Efficacy and safety of different doses of azilsartan medoxomil in patients with hypertension: A protocol of a network meta-analysis

**DOI:** 10.1097/MD.0000000000017050

**Published:** 2019-09-06

**Authors:** Yan Zhang, Huijin Yu, Kangmei Shao, Xinyue Luo, Jiancheng Wang, Gen Chen

**Affiliations:** a Spinal Cord Injury Rehabilitation Department, Rehabilitation Center Hospital of Gansu Province; b The Second Clinical Medical College of Lanzhou University; c Gansu Provincial Hospital; d Hospital Management Research Center, Lanzhou University; e Pathogens Biology Institute, School of Basic Medical Sciences, Lanzhou University, Lanzhou, China; f Basic medical school, Guilin Medical University, Guangxi, China.

**Keywords:** azilsartan medoxomil, hypertension, network meta-analysis

## Abstract

**Background:**

Hypertension is one of the most common chronic diseases and an increasingly public-health challenge worldwide. Previous meta-analyses evaluated the effects of azilsartan medoxomil compared to placebo or other antihypertensive drugs in patients with hypertension. However, it is still unclear which dose of azilsartan is optimal. This study will perform a network meta-analysis to assess the efficacy and safety of different doses of azilsartan medoxomil in patients with hypertension.

**Methods:**

PubMed, EMBASE.com, the Cochrane library, Scopus, and Web of Science were searched from inception to May 2019. Randomized controlled trials reporting efficacy and safety of different doses of azilsartan medoxomil on hypertension will be included if they compared 1 dose of azilsartan medoxomil with another dose of azilsartan medoxomil or with a placebo. Risk of bias of the included trials will be evaluated according to the Cochrane Handbook 5.1.0. NMA will be performed in a Bayesian hierarchical framework using WinBUGS 14.

**Results:**

The results will be submitted to a peer-reviewed journal for publication.

**Conclusion:**

This study will summarize all the available data to provide reliable evidence of the value of different doses of azilsartan medoxomil for the treatment of hypertension.

**PROSPERO registration number:**

CRD42019136882.

## 1 Introduction

Hypertension is a systemic disease characterized by elevated systemic arterial pressure, usually caused by the interaction of multiple genetics, environment, and various risk factors.^[1–3]^ It is one of the most common chronic diseases and an increasingly public-health challenge worldwide.^[4]^ Hypertension is also the most important risk factor for cardiovascular and cerebrovascular diseases, often accompanied by major complications, such as stroke, myocardial infarction, heart failure, and chronic kidney disease, which not only cause disability, high mortality but also severely consume medical and social resources, placing a heavy burden on families and countries.^[3,5–7]^

The underlying goal of hypertension treatment is to reduce the overall risk of development and death of heart, brain, kidney, and vascular complications. Commonly used antihypertensive drugs include calcium channel blockers (CCB), angiotensin-converting enzyme inhibitors (ACEI), angiotensin receptor blockers (ARB), diuretics, and beta blockers.^[8]^ ARB can reduce the incidence of cardiovascular complications in patients with a history of cardiovascular disease and the risk of cardiovascular events in hypertension patients, reducing proteinuria and microalbuminuria in patients with diabetes or kidney disease. ARB is especially suitable for patients with left ventricular hypertrophy, heart failure, diabetic nephropathy, coronary heart disease, metabolic syndrome, microalbuminuria or proteinuria, and patients who cannot tolerate ACEI.^[9–11]^ ARB drugs mainly include losartan, valsartan, irbesartan, telmisartan, candesartan, olmesartan, alisartan, and azilsartan.^[12,13]^ Compared with other ARBs, azilsartan binds tightly to AT1 and slowly separates. Furthermore, azilsartan induces insurmountable antagonism of angiotensin II-induced vascular contractions and inverse agonism against AT1.^[14–16]^ The high affinity and tight binding properties of azilsartan are expected to induce potent and long-lasting antihypertensive effects in preclinical and clinical settings.^[16–18]^

Recently, many meta-analyses evaluated the efficacy and safety of azilsartan medoxomil compared to placebo or other antihypertensive drugs in patients with hypertension.^[16,19,20]^ However, these studies are traditional meta-analyses that make it difficult to assess the effects of 2 or more interventions, although well-conducted systematic reviews of randomized controlled trials (RCTs) are often considered the best way to obtain evidence of healthcare decisions.^[21–23]^ Therefore, it is still unclear which dose of azilsartan is optimal. Network meta-analysis (NMA) allows for visualization of a larger amount of evidence, estimation of the relative effectiveness among all interventions, and rank ordering of the interventions even if some head to head comparisons are lacking.^[24,25]^ Thus, this study will perform a NMA to assess the efficacy and safety of different doses of azilsartan medoxomil in patients with hypertension.

## 2 Methods

The NMA will be conducted and reported in accordance with PRISMA extension version (PRISMA-NMA),^[26]^ and this protocol will be reported according to preferred reporting items for systematic review and meta-analysis protocols (PRISMA-P).^[27]^ The present protocol has been registered on the international prospective register of systematic review (PROSPERO) (CRD42019136882).

### 2.1 Search strategy

Experienced medical information experts worked with the review team to develop a comprehensive search strategy.^[28]^ A combination of subject terms and keywords was used and make appropriate adjustments of vocabulary and grammar between different databases. PubMed, EMBASE.com, the Cochrane library, SCOPUS, and Web of Science were searched from inception to May 2019. At the same time, the reference lists of published reviews and retrieved articles were checked for additional trials. The PubMed search strategy as follows:

#1 azilsartan kamedoxomil[Title/Abstract]
#2 azilsartan medoxomil[Title/Abstract]
#3 edarbi[Title/Abstract]
#4 ipreziv[Title/Abstract]
#5 tak 491[Title/Abstract]
#6 tak491[Title/Abstract]
#7 OR/1-6

### 2.2 Eligibility criteria

#### 2.2.1 Types of study

RCTs reporting efficacy and safety of different doses of azilsartan medoxomil on hypertension will be included if they compared 1 dose of azilsartan medoxomil with another dose of azilsartan medoxomil or with a placebo regardless of sample size. RCTs should report at least 1 measure outcome of efficacy and safety and provide enough detail to calculate effect sizes.

#### 2.2.2 Participants

Patients diagnosed with hypertension, regardless of age, gender, and duration of illness.

#### 2.2.3 Interventions

The intervention is the azilsartan medoxomil. The dose and duration of treatment are not limited.

#### 2.2.4 Comparators

The control can be another dose of azilsartan medoxomil or a placebo.

#### 2.2.5 Outcomes

The primary outcomes will include 24-hour mean diastolic blood pressure (DBP), 24-hour mean systolic blood pressure (SBP), nighttime DBP, nighttime SBP, daytime DBP, daytime SBP, clinic DBP, clinic SBP, and response rate. The second outcomes are any adverse events, serious adverse events, and adverse events lead to treatment interruption.

#### 2.2.6 Other criteria

The exclusion criteria are

(1) the intervention is azilsartan medoxomil combined with other antihypertensive drugs;
(2) comparison of 2 different types of antihypertensive drugs;
(3) nonrandomized trials, such as cohort study and observational study.

### 2.3 Selection of studies

The literature management software will be used to classify and organize the initial inspection documents, and to exclude duplicate documents. Then, 2 independent examiners will read the title and abstract of each record, and exclude studies that do not meet the inclusion criteria. For any potentially related study, we will review the full-text and determine the compliance studies based on the inclusion and exclusion criteria. The reasons for the excluded studies will be recorded. If we identify multiple similar articles published by the same author or institution, the one with long follow-up, the larger number of samples or more detailed data will be included, the remaining articles will be used as a supplement to the data. The intraclass correlation coefficient (ICC) will be used to assess the consistency of study selection between two reviewers.^[29]^ Disagreements will be discussed or by a third reviewer if no consensus is reached.

### 2.4 Data extraction

We will use predefined extraction forms with detailed written instructions to collect relevant information and data. Two independent reviewers will extract data from the included trials. The data extraction includes basic information such as first author, publication year, country, study time; information on hypertension; information on interventions (dose, time of treatment, follow-up time, etc); outcomes of interest. For studies did not report the data, we will contact the original author to get the relevant information. If there is a discrepancy between the two reviewers, a third researcher will be consulted.

### 2.5 Risk of bias assessment.

Two reviewers will independently use the Cochrane Handbook V.5.1.0 for systematic reviews of intervention to assess the quality of included RCTs. We will resolve any disagreement by discussion or by involving a third review author. The Handbook consists of random sequence generation, allocation concealment, blinding of all participants, including patients, personnel and outcome assessors, incomplete outcome data, selective reporting, and other sources of bias. We will rate the methodological quality as low, high or unclear risk of bias.

### 2.6 Statistical analysis

#### 2.6.1 Pairwise meta-analyses

For categorical variables, we will calculate the pooled odds ratio (OR) with the 95% confidence interval (95% CI). For continuous variables, we will calculate the standardized mean differences (SMDs) or mean differences (MDs) with 95% CIs. Study-specific estimates will be combined in the random-effects model.

We will assess statistical heterogeneity within each pairwise comparison using the *I*^2^ statistic. The values of 25%, 50%, and 75% for the *I*^2^ as indicative of low, moderate, and high statistical heterogeneity, respectively. We will explore sources of heterogeneity by subgroup analysis or meta-regression.

#### 2.6.2 Network meta-analysis

To assess the impact of azilsartan dosage on the pooled estimate, the effects of azilsartan therapy on blood pressure will be explored in the comparison of different doses of azilsartan with control therapy using a NMA. The NMA will be performed in a Bayesian hierarchical framework to incorporate both direct evidence and indirect evidence using Markov Chain Monte Carlo method in WinBUGS 14 (MRC Biostatistics Unit, Cambridge University, UK).^[30]^ The Brooks-Gelman-Rubin method will be used to assess convergence. We will compute a potential scale reduction factor (PSRF) by comparing within-chain and between-chain variance, and a PSRF very close to 1 is considered to indicate an approximate convergence.^[31]^ We will use the surface under the cumulative ranking curve (SUCRA) to rank the treatments according to each outcome accounting for the uncertainty in the treatment effects.^[32]^ The absolute ranks of the treatments per outcome are presented using “Rankograms” that visually show the distribution of ranking probabilities.^[33]^ The node splitting method will be adopted to examine the inconsistency between direct and indirect comparisons if a loop connecting 3 or more arms exist.^[32]^ A network plot will be plotted to describe and present the geometry of the treatment network of comparisons across trials to ensure if a NMA is feasible. The trials that are not linked by interventions will be excluded from the NMA, and we will just describe the findings of the study. All the result figures will be generated using STATA (13.0; Stata Corporation, TX,) software.

#### 2.6.3 Subgroup analysis and sensitivity analysis

If the necessary data are available, subgroup analyses will be done for different types of participants by gender and country. Sensitivity analyses will be performed to assess the contribution of each study to the pooled estimate by excluding individual trials 1 at a time and recalculating the pooled estimates for the remaining studies.

### 2.7 Assessment of publication bias.

Egger test and funnel plot will be conducted to detect the asymmetry due to publication bias when applicable.^[34,35]^

### 2.8 Quality of evidence

The Grading of Recommendations Assessment, Development and Evaluation (GRADE) tool will be used to assess the quality of evidence for each primary outcome. The GRADE approach assesses the quality of the body of evidence for each outcome considering 5 factors: study limitations, consistency of effect, indirectness, imprecision, and publication bias. The overall quality of evidence can be rated into 4 levels: high level, moderate level, low level, and very low level.^[36]^

## 3 Preliminary results

### 3.1 Study selection

According to the search strategy, related resources were retrieved, and 710 related records were obtained. After removing duplicates, 458 articles were obtained. Read the title and abstract to exclude 311 studies on non-hypertensive studies and concomitant medications, 15 news, errata, and drug information. Further screening of the remaining 132 full texts, excluding 3 pharmacokinetic studies, 17 studies comparing other antihypertensive drugs, 9 narrative reviews, 27 studies of combined drugs, and 6 systematic reviews or meta-analyses, 48 non-RCTs. Twenty-two studies were eventually included and 2 studies were supplemented by tracking references. Twenty-four studies reported 10 RCTs.

### 3.2 Main characteristics of some of the included studies

We extracted some data from the included RCTs, the main characteristics of some of the included studies are summarized in Table 1.

**Table 1:**
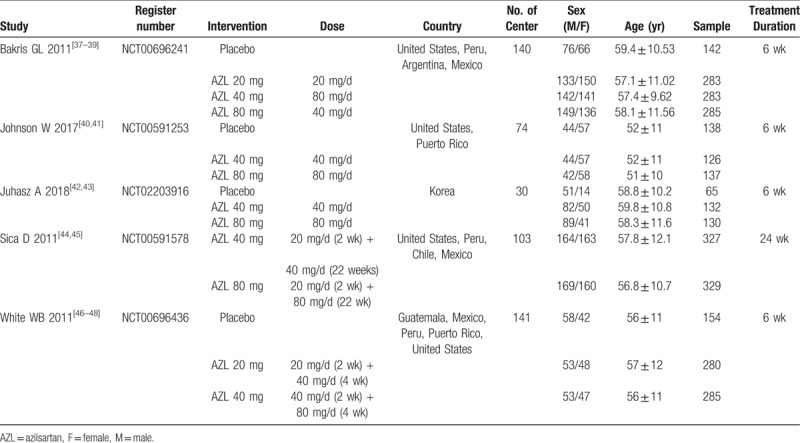
Main characteristics of some of the included studies.

## 4 Ethics and dissemination

Ethics approval and patient consent are not required as this study is a NMA based on published RCTs. This study will summarize all the available data to provide reliable evidence of the value of different doses of azilsartan medoxomil for the treatment of hypertension. The results will be submitted to a peer-reviewed journal for publication. We hope the findings of this NMA will help clinicians and patients choose the optimal dose of azilsartan medoxomil in treating hypertension.

## Author contributions

**Conceptualization:** Yan Zhang, Gen Chen.

**Data curation:** Yan Zhang, Huijin Yu, Xinyue Luo.

**Formal analysis:** Yan Zhang, Gen Chen.

**Funding acquisition:** Jiancheng Wang.

**Investigation:** Yan Zhang, Huijin Yu, Kangmei Shao, Xinyue Luo.

**Methodology:** Jiancheng Wang, Gen Chen.

**Project administration:** Gen Chen.

**Resources:** Yan Zhang, Huijin Yu, Kangmei Shao, Xinyue Luo, Gen Chen.

**Software:** Yan Zhang, Huijin Yu.

**Supervision:** Gen Chen.

**Validation:** Gen Chen.

**Visualization:** Kangmei Shao.

**Writing – original draft:** Yan Zhang, Huijin Yu, Gen Chen.

**Writing – review & editing:** Yan Zhang, Jiancheng Wang, Gen Chen.

## References

[1] Kjeldsen S Feldman RD Lisheng L Updated national and international hypertension guidelines: a review of current recommendations. Drugs 2014;74:2033–51. 25315030 10.1007/s40265-014-0306-5 PMC4224739

[2] Marques-Vidal P Arveiler D Amouyel P Sex differences in awareness and control of hypertension in France. J Hypertens 1997;15:1205–10. 9383168 10.1097/00004872-199715110-00003

[3] Valladares MJ Rodríguez Sándigo NA Rizo Rivera GO Prevalence, awareness, treatment, and control of hypertension in a small northern town in Nicaragua: The Elieth-HIFARI study. Health Sci Rep 2019;2:e120. 31346554 10.1002/hsr2.120 PMC6636515

[4] Bai Q Peng B Wu X Metabolomic study for essential hypertension patients based on dried blood spot mass spectrometry approach. IUBMB Life 2018;70:777–85. 30092118 10.1002/iub.1885

[5] Ganguly A Sharma K Majumder K Food-derived bioactive peptides and their role in ameliorating hypertension and associated cardiovascular diseases. Adv Food Nutr Res 2019;89:165–207. 31351525 10.1016/bs.afnr.2019.04.001

[6] Reklaitiene R Tamosiunas A Virviciute D Trends in prevalence, awareness, treatment, and control of hypertension, and the risk of mortality among middle-aged Lithuanian urban population in 1983–2009. BMC Cardiovasc Disord 2012;12:68. 22937997 10.1186/1471-2261-12-68 PMC3480954

[7] Tamosiunas A Klumbiene J Petkeviciene J Trends in major risk factors and mortality from main non-communicable diseases in Lithuania, 1985–2013. BMC Public Health 2016;16:717. 27492379 10.1186/s12889-016-3387-0 PMC4972981

[8] Redon J Weber MA Reimitz PE Comparative effectiveness of an angiotensin receptor blocker, olmesartan medoxomil, in older hypertensive patients. J Clin Hypertens (Greenwich) 2018;20:356–65. 29462508 10.1111/jch.13183 PMC8031060

[9] James PA Oparil S Carter BL 2014 evidence-based guideline for the management of high blood pressure in adults: report from the panel members appointed to the Eighth Joint National Committee (JNC 8). JAMA 2014;311:507–20. 24352797 10.1001/jama.2013.284427

[10] Weber MA Schiffrin EL White WB Clinical practice guidelines for the management of hypertension in the community: a statement by the American Society of Hypertension and the International Society of Hypertension. J Clin Hypertens (Greenwich) 2014;16:14–26. 24341872 10.1111/jch.12237 PMC8031779

[11] Mancia G Fagard R Narkiewicz K 2013 ESH/ESC guidelines for the management of arterial hypertension: the Task Force for the Management of Arterial Hypertension of the European Society of Hypertension (ESH) and of the European Society of Cardiology (ESC). Eur Heart J 2013;34:2159–219. 23771844 10.1093/eurheartj/eht151

[12] Angeli F Verdecchia P Pascucci C Pharmacokinetic evaluation and clinical utility of azilsartan medoxomil for the treatment of hypertension. Expert Opin Drug Metab Toxicol 2013;9:379–85. 23387516 10.1517/17425255.2013.769521

[13] Georgiopoulos G Katsi V Oikonomou D Azilsartan as a potent antihypertensive drug with possible pleiotropic cardiometabolic effects: a review study. Front Pharmacol 2016;7:235. 27536242 10.3389/fphar.2016.00235 PMC4971108

[14] Baker WL White WB Azilsartan medoxomil: a new angiotensin II receptor antagonist for treatment of hypertension. Ann Pharmacother 2011;45:1506–15. 22116996 10.1345/aph.1Q468

[15] Kurtz TW Kajiya T Differential pharmacology and benefit/risk of azilsartan compared to other sartans. Vasc Health Risk Manag 2012;8:133–43. 22399858 10.2147/VHRM.S22595 PMC3295635

[16] Takagi H Mizuno Y Niwa M A meta-analysis of randomized controlled trials of azilsartan therapy for blood pressure reduction. Hypertens Res 2014;37:432–7. 24108238 10.1038/hr.2013.142

[17] Handley A Lloyd E Roberts A Safety and tolerability of azilsartan medoxomil in subjects with essential hypertension: a one-year, phase 3, open-label study. Clin Exp Hypertens 2016;38:180–8. 26817604 10.3109/10641963.2015.1081213 PMC4819839

[18] Ojima M Igata H Tanaka M In vitro antagonistic properties of a new angiotensin type 1 receptor blocker, azilsartan, in receptor binding and function studies. J Pharmacol Exp Ther 2011;336:801–8. 21123673 10.1124/jpet.110.176636

[19] Zhang PX Ting S Feng XL Safety of azilsartan medoxomil in hypertension: a meta-analysis. J Am Coll Cardiol 2016;68:C140.

[20] Zhao D Liu H Dong P Antihypertensive effect of azilsartan versus olmesartan in patients with essential hypertension: a meta-analysis. Ir J Med Sci 2019;188:481–8. 29971568 10.1007/s11845-018-1859-1

[21] Ge L Tian JH Li YN Association between prospective registration and overall reporting and methodological quality of systematic reviews: a meta-epidemiological study. J Clin Epidemiol 2018;93:45–55. 29111471 10.1016/j.jclinepi.2017.10.012

[22] Tian JH Zhang J Ge L The methodological and reporting quality of systematic reviews from China and the USA are similar. J Clin Epidemiol 2017;85:50–8. 28063911 10.1016/j.jclinepi.2016.12.004

[23] Yao L Sun R Chen YL The quality of evidence in Chinese meta-analyses needs to be improved. J Clin Epidemiol 2016;74:73–9. 26780259 10.1016/j.jclinepi.2016.01.003

[24] Bafeta A Trinquart L Seror R Reporting of results from network meta-analyses: methodological systematic review. BMJ 2014;348:g1741. 24618053 10.1136/bmj.g1741 PMC3949412

[25] Gao Y Ge L Ma X Improvement needed in the network geometry and inconsistency of Cochrane network meta-analyses: a cross-sectional survey. J Clin Epidemiol 2019;113:214–27. 31150834 10.1016/j.jclinepi.2019.05.022

[26] Hutton B Salanti G Caldwell DM The PRISMA extension statement for reporting of systematic reviews incorporating network meta-analyses of health care interventions: checklist and explanations. Ann Intern Med 2015;11:777–84. 10.7326/M14-2385 26030634

[27] Shamseer L Moher D Clarke M Preferred reporting items for systematic review and meta-analysis protocols (PRISMA-P): 2015: elaboration and explanation. BMJ 2015;349:g7647. 10.1136/bmj.g7647 25555855

[28] Li L Tian J Tian H Network meta-analyses could be improved by searching more sources and by involving a librarian. J Clin Epidemiol 2014;67:1001–7. 24841794 10.1016/j.jclinepi.2014.04.003

[29] Yan P Yao L Li H The methodological quality of robotic surgical meta-analyses needed to be improved: a cross-sectional study. J Clin Epidemiol 2019;109:20–9. 30579979 10.1016/j.jclinepi.2018.12.013

[30] Lunn DJ Thomas A Best N WinBUGS-A Bayesian modeling framework: concepts, structure, and extensibility. Stat Comput 2000;10:325–37.

[31] Zhang Z Hou Y Zhang J Comparison of the effect of oral care with four different antiseptics to prevent ventilator-associated pneumonia in adults: protocol for a network meta-analysis. Syst Rev 2017;6:103. 28526060 10.1186/s13643-017-0496-5 PMC5437639

[32] Lu G Ades AE Combination of direct and indirect evidence in mixed treatment comparisons. Stat Med 2004;23:3105–24. 15449338 10.1002/sim.1875

[33] Salanti G Indirect and mixed-treatment comparison, network, or multiple treatments meta analysis: many names, many benefits, many concerns for the next generation evidence synthesis tool. Res Synth Methods 2012;3:80–97. 26062083 10.1002/jrsm.1037

[34] Begg CB Mazumdar M Operating characteristics of a rank correlation test for publication bias. Biometrics 1994;50:1088. 7786990

[35] Egger M Davey Smith G Schneider M Bias in meta-analysis detected by a simple, graphical test. BMJ 1997;315:629–34. 9310563 10.1136/bmj.315.7109.629 PMC2127453

[36] Puhan MA Schünemann HJ Murad MH A GRADE Working Group approach for rating the quality of treatment effect estimates from network meta-analysis. BMJ 2014;349:g5630. 25252733 10.1136/bmj.g5630

[37] Bakris G Sica D Weber M Results of a novel angiotensin receptor blocker, azilsartan medoxomil, in patients with primary hypertension. J Hypertens 2010;28:E429.

[38] Bakris GL Sica D Weber M The comparative effects of azilsartan medoxomil and olmesartan on ambulatory and clinic blood pressure. J Clin Hypertens (Greenwich) 2011;13:81–8. 21272195 10.1111/j.1751-7176.2010.00425.x PMC8673073

[39] Bakris G Sica D Weber M Effects of the novel angiotensin receptor blocker azilsartan medoxomil in patients with primary hypertension. J Clin Hypertens (Greenwich) 2011;12:A106–7.

[40] Johnson W White WB Sica D Efficacy and safety of azilsartan medoxomil, a novel angiotensin ii receptor blocker, in African–Americans with hypertension. J Clin Hypertens (Greenwich) 2010;12:A111.

[41] Johnson W White WB Sica D Evaluation of the angiotensin II receptor blocker azilsartan medoxomil in African-American patients with hypertension. J Clin Hypertens (Greenwich) 2017;19:695–701. 28493376 10.1111/jch.12993 PMC8031359

[42] Juhasz A Wu J Hisada M Efficacy and safety of azilsartan medoxomil, an angiotensin receptor blocker, in Korean patients with essential hypertension. Clin Hypertens 2018;24:2. 29445520 10.1186/s40885-018-0086-4 PMC5804062

[43] Juhasz A Wu J Hisada M Efficacy and safety of the novel angiotensin receptor blocker, azilsartan-medoxomil, in korean patients with essential hypertension. J Hypertens 2016;34:E84–5. 10.1186/s40885-018-0086-4 PMC5804062 29445520

[44] Sica D White WB Weber MA New angiotensin II receptor blocker azilsartan medoxomil: comparison to valsartan. J Hypertens 2010;28:E276.

[45] Sica D White WB Weber MA Comparison of the novel angiotensin II receptor blocker azilsartan medoxomil vs valsartan by ambulatory blood pressure monitoring. J Clin Hypertens (Greenwich) 2011;13:467–72. 21762358 10.1111/j.1751-7176.2011.00482.x PMC8108745

[46] White WB Weber MA Sica D The new angiotensin receptor blocker - Azilsartan medoxomil has significantly greater 24-hour blood pressure lowering efficacy to both olmesartan and valsartan. J Hypertens 2010;28:E442.

[47] White WB Weber MA Sica D Effects of the angiotensin receptor blocker azilsartan medoxomil versus olmesartan and valsartan on ambulatory and clinic blood pressure in patients with stages 1 and 2 hypertension. Hypertension 2011;57:413–20. 21282560 10.1161/HYPERTENSIONAHA.110.163402

[48] White WB Weber MA Sica D The new angiotensin receptor blocker azilsartan medoxomil has superior 24hour blood pressure lowering efficacy to both olmesartan and valsartan. J Hypertens 2010;12:A116.

